# Novel Antibacterial Resin Coating for Dental Provisional Crowns to Suppress Biofilms and Inhibit Secondary Caries

**DOI:** 10.3390/coatings14111370

**Published:** 2024-10-28

**Authors:** Ibrahim Ba-Armah, Mohammad Alenizy, Nader Almutairi, Heba Alqarni, Abdullah Alhussein, Radi Masri, Gary D. Hack, Thomas W. Oates, Jirun Sun, Michael D. Weir, Hockin H. K. Xu

**Affiliations:** 1 Dental Biomedical Sciences PhD Program, Graduate School, University of Maryland, Baltimore, MD 21201, USA; 2 Department of Biomaterials and Regenerative Dental Medicine, University Maryland School of Dentistry, Baltimore, MD 21201, USA; 3 Department of Restorative Dental Science, College of Dentistry, Imam Abdulrahman Bin Faisal University, Dammam 31441, Saudi Arabia; 4 Department of Restorative Dental Sciences, College of Dentistry, University of Hail, Hail 55475, Saudi Arabia; 5 Department of Conservative Dental Sciences, College of Dentistry, Prince Sattam bin Abdulaziz University, Alkharj 16245, Saudi Arabia; 6 Department of Pediatric Dentistry and Orthodontics Sciences, College of Dentistry, King Khalid University, Abha 61421, Saudi Arabia; 7 Department of Restorative Dental Science, College of Dentistry, King Saud University, Riyadh 11545, Saudi Arabia; 8 Department of Advanced Oral Sciences and Therapeutics, University Maryland School of Dentistry, Baltimore, MD 21201, USA; 9 The ADA Forsyth Institute, Cambridge, MA 02142, USA; 10 Center for Stem Cell Biology & Regenerative Medicine, University of Maryland School of Medicine, Baltimore, MD 21201, USA; 11 Marlene and Stewart Greenebaum Cancer Center, University of Maryland School of Medicine, Baltimore, MD 21201, USA

**Keywords:** provisional crowns, resin, coating, antibacterial, secondary caries

## Abstract

Provisional crowns are often used in dentistry for prolonged periods, but bacterial attachment and dental plaque often lead to gingival inflammation and secondary caries. The aims of this research were to develop a novel resin-based antibacterial provisional crown coating to prevent secondary caries and investigate the physical properties and antibacterial efficacy. The resin-based coating was prepared by addition of triethylene glycoldivinylbenzyl ether and urethane dimethacrylate, with the antibacterial monomer dimethylaminododecyl methacrylate (DMADDM) incorporated at different mass fractions. Surface characteristics including surface roughness and contact angle were assessed. The antibacterial effects were evaluated by 48 h biofilms of *Streptococcus mutans* (*S. mutans*) on provisional crowns coated with the resin-based coating. No statistically significant difference was observed in surface roughness across all groups (*p* > 0.05), showing that adding DMADDM did not have a negative impact on surface roughness. The contact angle results revealed a significant difference in hydrophilicity between different concentrations of DMADDM (*p* < 0.01), but overall hydrophilicity did not negatively affect the performance of the coating. The incorporation of 5% DMADDM demonstrated a significant antibiofilm effect on *S. mutans* biofilm CFU with a 4-log reduction compared to controls (*p* < 0.01). Significant reductions of 4–5 folds were observed in biofilm metabolic activity and lactic acid production (*p* < 0.01). The findings suggest that the novel coating material could enhance the long-term performance and clinical outcomes of provisional crowns, contributing to better patient oral health.

## 1. Introduction

Temporary dental prostheses or provisional crowns are an essential part and crucial for the success of fixed prosthetic treatments. In clinical scenarios that necessitate modifications to the occlusal vertical or horizontal dimensions, provisional crowns may remain in service for prolonged phases up to two years [1–4]. Provisional crowns serve the objective of maintaining tooth function, acceptable speech, smile appearance, and gingival tissue health [5–7]. They also ensure tissue compatibility until the final prostheses are made. Furthermore, they can also contribute to the contouring of marginal gingival regions. Provisional crowns are utilized to shield the treated teeth from chemical, thermal, and bacterial irritations. Additionally, they might be employed to visualize the intended design of the final crowns [8–10]. Most provisional crowns are fabricated with resin-based materials. These materials are correlated with several limitations that result in unfavorable conditions, particularly in prolonged utilization periods [11]. Generally, provisional crowns have weak mechanical properties, high surface roughness values, and inferior fitting interfaces accompanied with lower marginal adaptability, leading to increased risk of plaque deposition, secondary caries, and periodontal disease [12–15].

The long-term performance of provisional crowns in the clinical setting remains contingent upon their accurate construction. However, it remains challenging to create crown restorations that fit perfectly around the margins of the tooth structure [16]. The marginal gaps around provisional crowns make them more susceptible to microleakage, facilitating bacterial infiltration, retention, and colonization. Acids generated by these bacteria can cause demineralization and secondary caries [17,18].

The rougher surfaces of provisional crowns are more prone to bacterial adherence compared to the materials used for final restorations. The relationship between the surface roughness of dental materials and bacterial adhesion has been previously established [19]. In most cases, the smoothness of crown surfaces can be enhanced through polishing techniques. Failure to follow polishing protocols increases the risk of bacterial adhesion due to critical roughness. Surface irregularities, such as pits and grooves, on rough surfaces increase bacterial adherence by reducing shear forces and protecting bacteria from salivary flow and masticatory actions, thus promoting adhesion to more sites on the surfaces [20–22].

Additionally, hydrophilic surfaces attract and absorb tissue fluids, such as blood, gingival crevicular fluid, and saliva, causing attachment proteins like fibronectin and mucins to be adsorbed. Although numerous cells involved in tissue regeneration utilize these proteins for attachment to the prosthesis, these proteins may similarly contain binding sites for bacteria [23–25]. The microtopography and hydrophilicity of the surfaces have an impact on the initial development and composition of the biofilm [25]. Moreover, the quantity of bacteria that attach to a particular surface is influenced by certain surface characteristics, including hydrophobicity and surface charge. Moreover, the bacterial adhesion process is significantly impacted by the surface hydrophobicity of a substance, in addition to its chemical composition. Typically, microorganisms that are hydrophobic prefer materials that are hydrophobic, while bacteria that possess hydrophilic properties prefer materials that are hydrophilic [26,27]. Oral bacteria attach to both hydrophobic and hydrophilic surfaces; however, because of varying shear forces, minimal biofilm forms on hydrophobic surfaces in vivo [28].

One of the main cariogenic pathogens in caries development is *Streptococcus mutans* (*S. mutans*) [29]. *S. mutans*’ cariogenic potential is mostly related to its power to make organic acids by carbohydrate metabolism, its capability to manufacture glucosyltransferases which synthesis extracellular polysaccharides, and its power of surviving under low-pH environments [30–32].

Quaternary ammonium methacrylates (QAMs) have promising potential clinical applications showing potent antibacterial effects against dental biofilms [33,34]. Recently, dimethylaminododecyl methacrylate (DMADDM) was formulated, providing long-term antibacterial properties without affecting the mechanical characteristics and biocompatibility of dental materials [35]. DMADDM can promote bacterial killing by its interaction with the positively charged quaternary amine and the negatively charged cell membrane, yielding a contact-killing antimicrobial effect by which the cell membrane loses its electrical balance, and eventually the bacterial membrane rupture under their own osmotic pressure, due to damage of the cytoplasmic membrane of bacteria, releasing cytoplasmic constituents. Studies have confirmed these findings, demonstrating that QAMs can cause cell lysis and death by damaging both the outer and cytoplasmic membranes [36,37]. DMADDM has been incorporated in various dental materials, like denture base materials, dental implants coatings, and resin-based dental composites, to form a bioactive dental material [38–40].

Triethylene glycol divinylbenzyl ether (TEG-DVBE) is a low viscosity ether-based monomer capable of withstanding hydrolysis conditions. When combined with urethane dimethacrylate (UDMA), it slows down polymerization, causing a delayed gel phase. The (TEG-DVBE UDMA) resin exhibits a high degree of conversion and a flexible, hydrophobic structure, making it effective in withstanding hydrolytic and enzymatic degradation, minimizing water absorption, and reducing stress from polymerization shrinkage [41,42].

Recently, studies showed promising physical, mechanical, and antibacterial properties by incorporating DMADDM into resin composite, pit and fissure sealant, dental primers, and denture base resins [33,34,39,40]. Several studies also investigated the effect of combining TEG-DVBE UDMA resin with QAMs into low-shrinkage resin composites, resin-based cements, and root surface coatings [42–44]. However, no studies have attempted to incorporate DMADDM into TEG-DVBE UDMA resin to develop a resin-based provisional crown coating.

Therefore, in this research, the aims were to develop a novel resin-based provisional crown coating modified with DMADDM to inhibit secondary caries around the gingival margins of provisional crowns and investigate its antibacterial efficacy. The following hypotheses were tested: (1) the novel resin-based provisional crown coating containing DMADDM will significantly reduce *Streptococcus mutans*’ bacterial viability, lactic acid production, and metabolic activity compared to the commercial control; (2) the novel resin-based coating will not negatively impact the viability of human gingival fibroblast cells; and (3) the surface characteristics of the novel resin-based coating with DMADDM will be comparable to those of the commercial control.

## 2. Materials and Methods

### 2.1. Synthesis of the Resin-Based Coating

The resin matrix for the resin-based coating, designated as “UV” resin, was prepared by the addition of 55.8% UDMA (Esstech, Essington, PA, USA) and 44.2% TEG-DVBE (all mass %), as determined in earlier studies [35]. Photoinitiators comprising 0.8% of 4-N, N-dimethylaminobenzoate (4EDMAB; MilliporeSigma, Burlington, MA, USA), and 0.2% camphorquinone (CQ, MilliporeSigma, Burlington, MA, USA) were utilized. DMADDM was produced by a modified Menschutkin reaction by mixing tertiary amines with organohalides. A solution of 10 mmol of 1-bromododecane (BDD) (TCI America, Portland, OR, USA), 10 mmol of 2-(dimethylamino) ethyl methacrylate (DMAEMA, Aldrich, St. Louis, MO, USA), and 3 g of ethanol as the solvent was agitated in a glass container for 24 h at 70 °C to generate DMADDM. Upon evaporation of the ethanol, the resulting DMADDM was a waxy white solid. DMADDM was incorporated in the UV resin at concentrations of 2.5%, 5%, 7.5%, and 10% by final mass. In this study, TEMPSMART^®^ (GC America Inc., Alsip, IL, USA) was designated as a commercial control. It is a dual-cured, bis-acrylic composite for temporary crown material.

Then, the following resin-based coatings were developed:

1. TEMPSMART (GC America) (denoted as “Commercial Control”);
2. UV resin + 0% DMADDM (denoted as “Experimental Control”);
3. UV resin + 2.5% DMADDM (denoted as “UV + 2.5% DMADDM”);
4. UV resin + 5% DMADDM (denoted as “UV + 5% DMADDM”);
5. UV resin + 7.5% DMADDM (denoted as “UV + 7.5% DMADDM”);
6. UV resin + 10% DMADDM (denoted as “UV + 10% DMADDM”).

### 2.2. Samples Preparation and Analysis of Surface Roughness

Disk-shaped specimens were created using the commercial TEMPSMART provisional crown composites to have a diameter of 8 mm and a thickness of 1 mm. The samples were light-cured for 90 s (Labolight DUO, GC America, Alsip, IL, USA) from each surface while being covered with clear matrix to inhibit the development of an air-inhibited layer and pressed between two microscope slides to provide a flat surface. Disks were then randomly divided between the six groups. Using a plastic transfer pipette, an equal amount of the UV resin-based coating (20 ± 2 mg) was dropped on each surface of the provisional crown disk corresponding to the five experimental groups. The coated surface was covered with clear matrix and then light cured for 60 s. The coating process was repeated for each surface of the disks. For the Commercial Control group, the disks were left uncoated. Each specimen was polished using wet abrasive paper disks (Buehler’s CarbiMet, Buehler Ltd., Lake Bluff, IL, USA). The Commercial Control group was finished and subsequently polished with a grit of 400, 600, 800, 1000, 1200, and 2000. All other specimens with resin-based coating were only polished with 1000 and 1200 grits. The mean value of surface roughness (Ra, μm) was determined using a surface roughness analyzer (Surftest SJ-310; Mitutoyo America, Aurora, IL, USA). A stylus with a tip radius of 5μm was used to traverse each specimen at a consistent rate of 0.5 mm/s, a force of 4 mN, and a cutoff value of 0.25 mm, with a trace length of 1.5 mm.

### 2.3. Contact Angle Assays

The water contact angle was obtained to determine the hydrophilicity of the resin disk specimens. Using the contact angle measurement apparatus (Dropometer, Droplet Lab, Markham, ON, Canada), the water contact angle was measured via the sessile-drop technique in air [45]. A total of 15 measurements were taken. Next, 5 μL of deionized (DI) water droplets was applied onto resin disks, and the contact angle was evaluated during a 10 s timeframe.

The hydrophilicities of the uncured UV resin-based coatings were analyzed by contact angle measurements (n = 15). A standard 3 μL droplet of each of the five experimental groups of UV resin was individually applied onto the surface of the TEMPSMART resin disk. After 10 s, an image of the droplet’s shape was captured using the apparatus. The Droplet Lab’s Sessile software (version 1.0.5.1) yielded the contact angle data by the utilization of the Young–Laplace equation [45].

### 2.4. Resin-Based Coating Thickness

The thicknesses of the UV resin-based coatings were evaluated by a scanning electron microscope (Quanta 200, FEI Company, Hillsboro, OR, USA). One specimen from each experimental group was split into two halves using a straight handpiece with a diamond disk. Each specimen was mounted vertically, showing the interface between the UV resin coating and the provisional resin disks. Following that, the specimens were coated with platinum using sputter coating and analyzed using SEM. The UV resin-based coating thicknesses were obtained by analyzing SEM images. The average thickness was calculated out of five measurements from each specimen.

### 2.5. Samples Preparation for S. mutans Biofilm Testing

Resin disks (n = 6) were prepared similar to the surface roughness testing specimens mentioned previously without the finishing and polishing steps. All specimens were placed in an incubator for 24 h at 37 °C. After 24 h, the specimens were kept in DI water and agitated for 1 h at 100 rpm to eradicate unpolymerized monomers. The disk specimens (n = 6) from each group underwent sterilization with ethylene oxide (Anprolene AN 74i, Andersen, Haw River, NC, USA). Specimens were placed in a desiccator for 7 days to facilitate the elimination of residual ethylene oxide, in accordance with the manufacturer’s directions.

### 2.6. Inoculation of S. mutans and Biofilm Formation

Utilization of bacterial species was permitted by the Institutional Review Board of the University of Maryland, Baltimore, MD, USA (HP-00052180). *S. mutans* (UA159) was selected due to its significant correlation with dental caries. A strain of *S. mutans* was cultivated overnight in brain heart infusion (BHI) broth (Sigma-Aldrich, St. Louis, MO, USA) at a temperature of 37 °C with 5% CO_2_, which was consistent for all biofilm experiments. To achieve an inoculum density of 107 colony-forming units (CFU) per milliliter, a spectrophotometer (Genesys 10S, ThermoScientific, Waltham, MA, USA) was employed, using the standard curve of OD600 nm versus CFU/mL. The disk specimens were transferred into a 24-well plate and submerged in a 1.5 mL of BHI culture medium with 2% sucrose (by weight/volume). Samples were then incubated for 24 h. Then, the disk specimens were moved to different 24-well plates and submerged in 1.5 mL of fresh BHI media with sucrose. Subsequently, they were incubated for a further 24 h to complete a 48-h total incubation period under static conditions. The 48 h incubation period was determined to be adequate for the development of mature biofilms on resin specimens.

### 2.7. S. mutans Biofilm Colony-Forming Units (CFU)

The biofilms on resin specimens (n = 6) that develop over a two-day period were collected on a new 24-well plate with 1 mL of PBS by scraping and sonication. Suspensions were diluted in a series of 10^1^–10^6^-fold and then dispersed on BHI agar plates. The plates were then incubated for 48 h at 37 °C in 5% CO_2_. The colony-forming unit (CFU) counts were calculated by recording the number of colonies after 48 h. Three replicates of the CFU tests were conducted.

### 2.8. Metabolic Activity of S. mutans Biofilms (MTT)

The biofilm’s metabolic activity was evaluated by colorimetric analysis with 3-[4,5-dimethylthiazol-2-yl]-2,5-diphenyltetrazolium bromide (MTT). Biofilm-containing disk specimens were moved to a new 24-well plate and exposed to 1 mL of MTT dye solution (0.5 mg/mL MTT in PBS). Subsequently, the plate was placed in an incubator for 1 h at 37 °C and 5% CO_2_. The specimens were then moved to a different plate and covered with 1 mL of dimethyl sulfoxide (DMSO) and then kept in a dark area at room temperature for 20 min to suspend the formazan crystals. A 200 μL amount of the DMSO solution was extracted from each specimen and transferred to a 96-well plate in order to quantify the absorbance. Using a microplate reader (SpectraMax M5, Molecular Devices, Sunnyvale, CA, USA), absorbance was measured at a wavelength of 540 nm. A higher absorbance value implies a higher concentration of formazan, therefore indicating a higher metabolic activity of the biofilm on the disk. MTT experiments were conducted three times.

### 2.9. Lactic Acid Production by S. mutans Biofilms

Resin disks covered by 48 h biofilms were transferred to 24-well plates filled with buffered peptone water (BPW) with 0.2% sucrose and placed in the incubator at 37 °C in 5% CO_2_ for a duration of 3 h. In order to assess lactate concentrations in BPW, the optical density was determined at 340 nm using a microplate reader (Spectra-Max M5, Molecular Devices, Sunnyvale, CA, USA) through an enzymatic test involving lactate dehydrogenase. This was done following procedures that were previously described [46]. The experiment of lactic acid production was conducted three times.

### 2.10. Live/dead Staining of Biofilm

Resin specimens covered with biofilm were rinsed with phosphate-buffered saline (PBS) to eliminate unattached bacteria. The specimens underwent staining with the BacLight live/dead kit (Molecular Probes, Eugene, OR, USA). Each specimen was submerged for a duration of 15 min in a solution containing 2.5 μM SYTO 9 and 2.5 μM propidium iodide. The live bacteria were stained with SYTO9, resulting in the emission of green fluorescence. Bacteria with disrupted membranes became stained with propidium iodide, which caused them to emit red fluorescence. A Fluorescent microscope (BioTek Cytation 5, Agilent Technologies, Santa Clara, CA, USA) was used to evaluate the biofilms on the specimens.

### 2.11. Scanning Electron Microscopy (SEM) of S. mutans Biofilms

The resin disks with 48 h biofilms were rinsed with PBS and covered in 4% paraformaldehyde at 4 °C overnight. Afterwards, specimens were washed with PBS and desiccated using a sequence of ethanol solutions. The specimens were then left to desiccate overnight. A platinum sputter coating was applied to the surface of the specimens. Biofilms on resin disks were visualized using a scanning electron microscope (Quanta 200, FEI Company, Hillsboro, OR, USA).

### 2.12. Cytotoxicity of Human Gingival Fibroblasts

Human gingival fibroblast cells (CRL-4061 TERT, ATCC) were used to assess cytotoxicity. Fibroblast medium (FM) (Sciencell Research Laboratories, Carlsbad, CA, USA) used in this study contained 1% by weight of fibroblast growth supplement, 2% by weight of fetal bovine serum, and 10,000 units/mL of penicillin and 10,000 μg/mL of streptomycin. The process of cell seeding was initiated once the cell viability had surpassed 90%. Cells were distributed in a 96-well plate at a density of 5000 cells per well and then placed for a 24 h in an incubator at a temperature of 37 °C with 5% CO_2_. Three resin-coated specimens, shaped like disks with a 4 mm diameter and 1 mm thickness, were sterilized using the same procedure detailed earlier in the biofilm testing preparation. Subsequently, specimens were immersed in 4 mL of medium and incubated for 24 h at 37 °C. The calculated surface area to solution ratio was 0.63 cm^2^/mL, falling within the acceptable range of 0.5–6 cm^2^/mL as defined by ISO 10993–12:2021. Subsequently, 100 μL of the initial extracts from each sample were introduced into grown cells and then incubated for 24 h at 37 °C with 5% CO_2_. After that, 10 μL of a substrate solution containing water-soluble tetrazolium salts (cell counting kit-8, Dojindo, Rockville, MD, USA) was added to each well and placed in the incubator for 2 h under identical circumstances. The degree of cellular dehydrogenase activity in the culture medium was assessed by measuring the absorbance at 450 nm. The negative control for the human gingival fibroblasts (HGFs) was a culture medium without any extracts. The cell viability experiment was conducted in triplicate.

### 2.13. Statistical Analyses

All the statistical analyses, including normality assessment via the Shapiro–Wilk test, were conducted using SPSS 19.0 software (SPSS, Chicago, IL, USA). A one-way analysis of variance (ANOVA) was used to identify statistically significant associations between the variables. A Tukey multiple comparison test was used to compare the available data. Probability of significance was established at a *p*-value of <0.05.

## 3. Results

### 3.1. Surface Roughness

**Figure 1.**
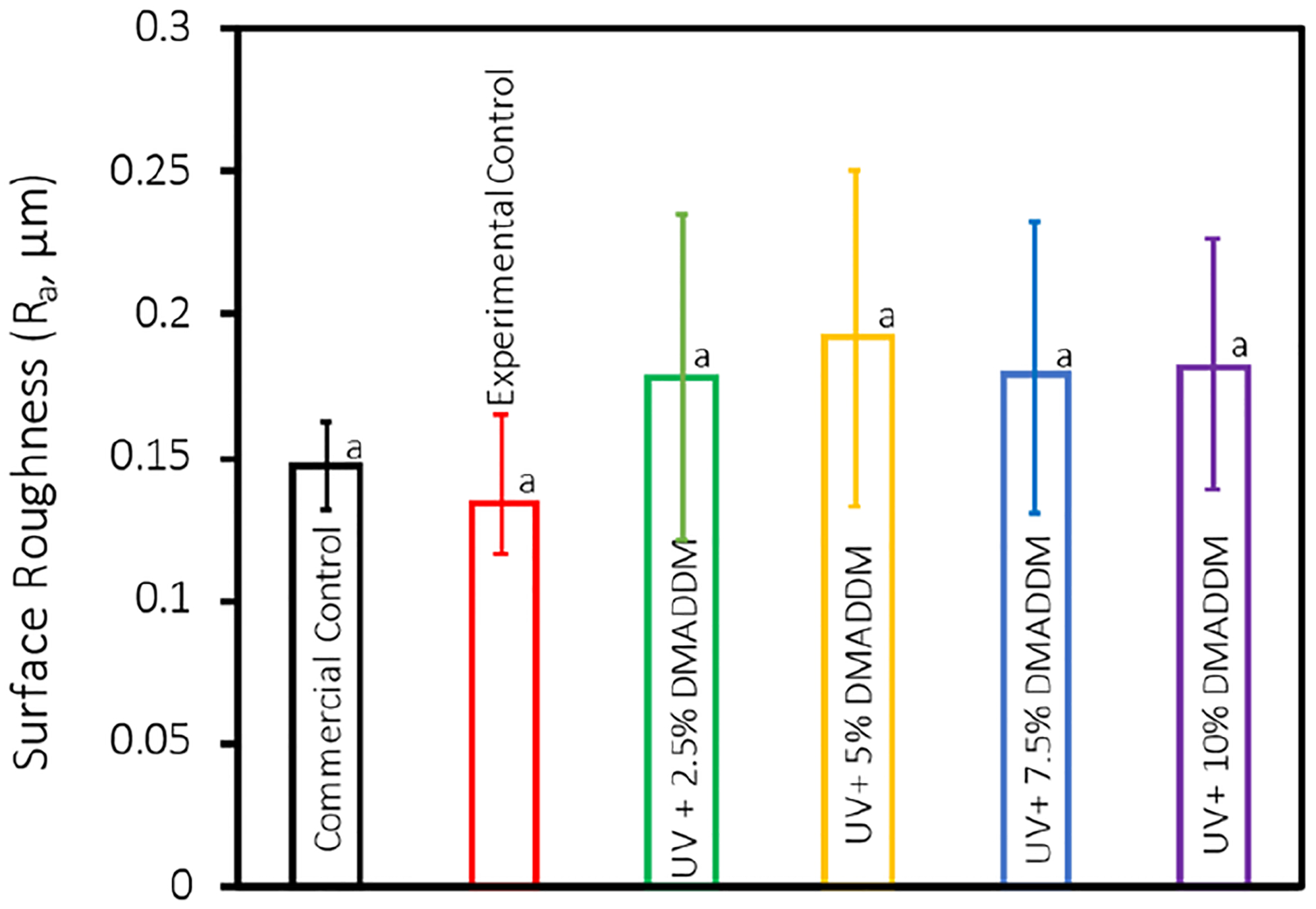
Surface roughness values for each group (mean ± sd; n = 10). No significant difference in surface roughness values was found between Commercial Control and resin-based coatings with different DMADDM concentrations (*p* > 0.05). Data with significant differences (*p* < 0.01) are indicated by different letters.

The results of surface roughness for each group are shown in Figure 1 (mean ± sd; n = 10). No significant differences in surface roughness values were found between Commercial Control and resin-based coatings with different DMADDM concentrations (*p* > 0.05), showing that adding DMADDM did not adversely affect surface roughness.

### 3.2. Contact Angle Assays

**Figure 2.**
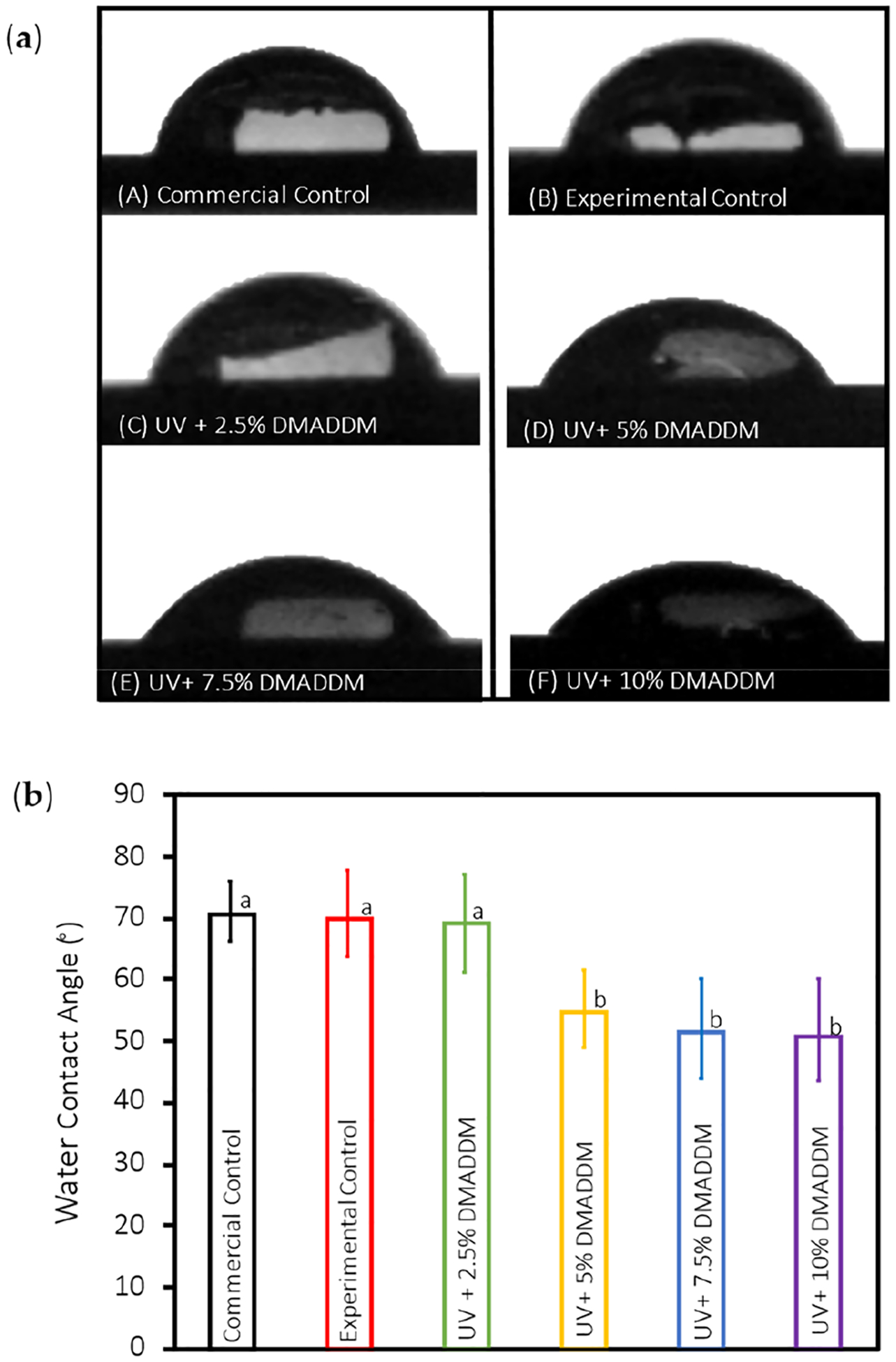
Water contact angle of the samples: (**a**) the representative images of water droplets on the samples; (**b**) the statistical analysis of the water contact angle (mean ± sd; n = 15). Data with significant differences (*p* < 0.01) are indicated by different letters.

For wettability assessment, the water contact angle results are shown in Figure 2 (mean ± sd; n = 15). The Commercial Control group had a contact angle of (70.5 ± 4.6)°, the Experimental Control group’s contact angle was (70 ± 6.8)°, and the UV+ 2.5% DMADDM group had a contact angle of (69.1 ± 7.6)°, with no significant difference between them (*p* > 0.01). The contact angle of UV+ 5% DMADDM was (54.7 ± 5.9)°, the UV+ 7.5% DMADDM had a contact angle of (51.7 ± 7.6)°, and the UV+ 10% DMADDM had a contact angle of (50.7 ± 8)°, with no significant difference between them (*p* > 0.01). However, there was a significant difference between the Commercial Control, Experimental Control and UV + 2.5% DMADDM groups and the UV+ 5% DMADDM, UV+ 7.5% DMADDM, and UV+ 10% DMADDM groups (*p* < 0.01).

**Figure 3.**
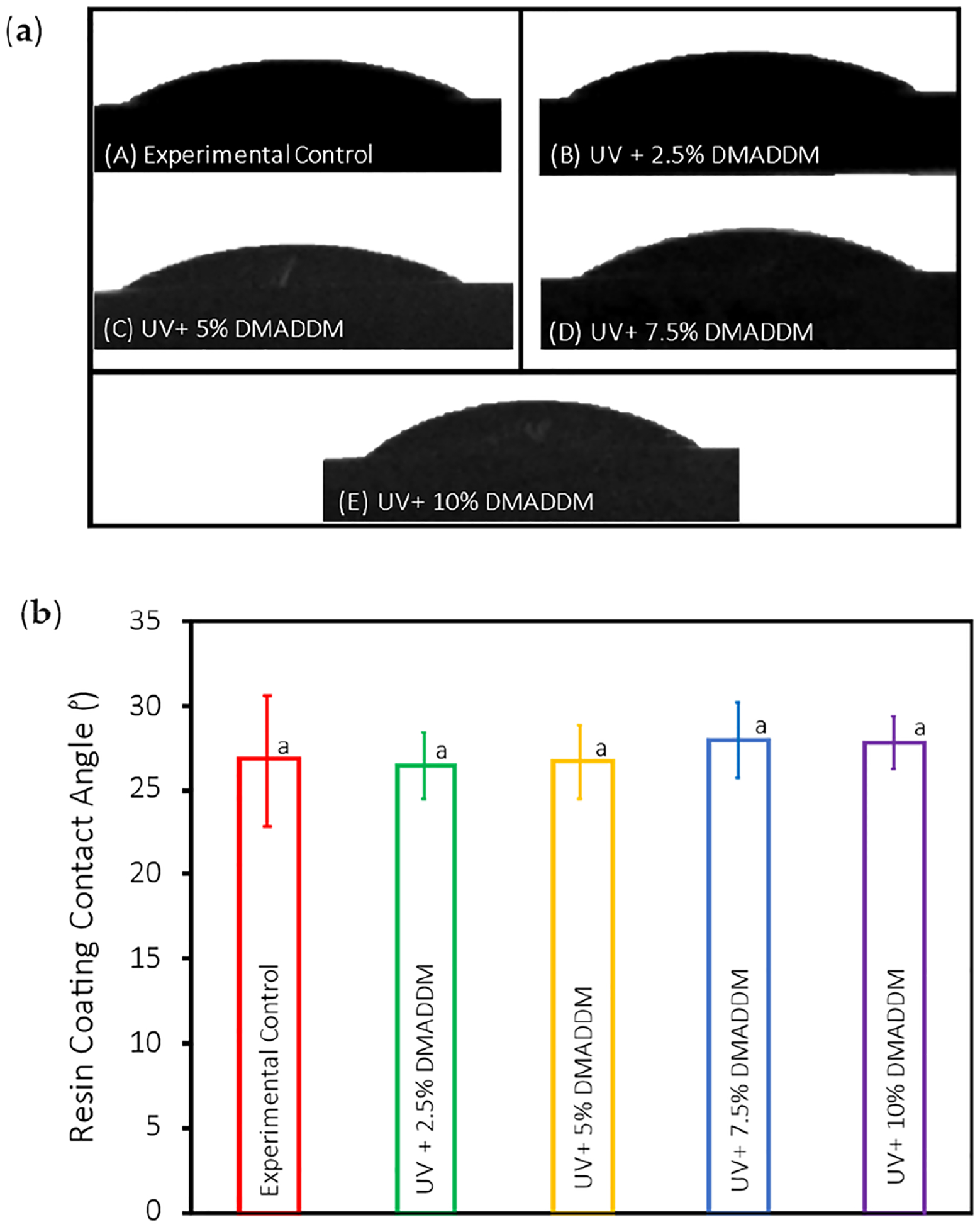
Contact angle of the uncured UV resin-based coatings: (**a**) The representative images of uncured resin coating droplets on the samples; (**b**) The statistical analysis of the resin coating contact angle (mean ± sd; n = 15). Data with significant differences (*p* < 0.01) are indicated by different letters.

The contact angle results for uncured UV resin-based samples are presented in Figure 3 (mean ± sd; n = 15). The Experimental Control group’s contact angle was (26.84 ± 3.8)° and the UV+ 2.5% DMADDM group had a contact angle of (26.48 ± 1.9)°, the contact angle of the UV+ 5% DMADDM was (26.70 ± 2.1)°, the UV+ 7.5% DMADDM had a contact angle of (27.95 ± 2.1)°, and the UV+ 10% DMADDM had a contact angle of (27.84 ± 1.5)°, with no significant difference between all the groups (*p* > 0.01).

### 3.3. SEM Analysis of Resin-Based Coating Thickness

**Figure 4.**
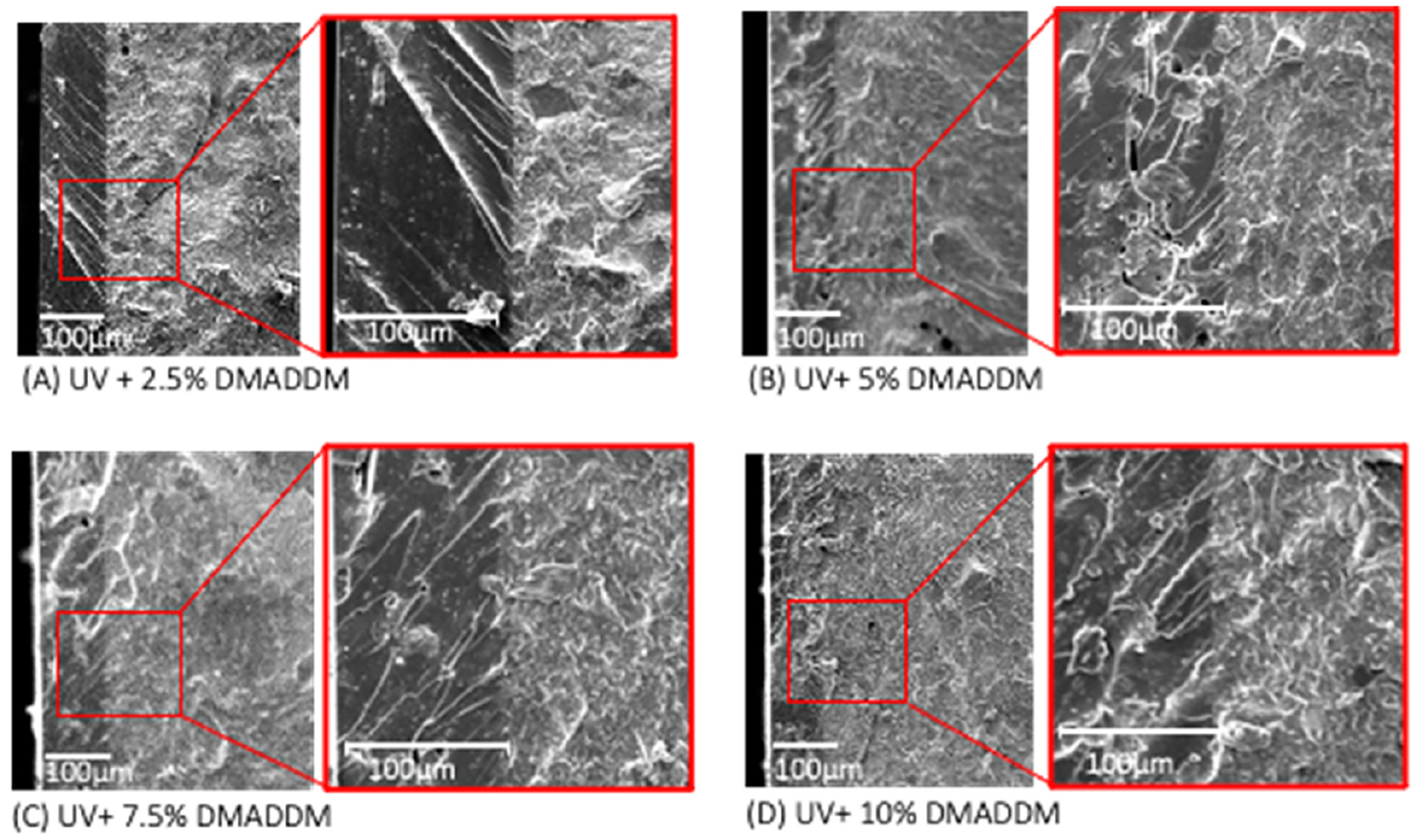
SEM images of the UV resin-based coating containing DMADDM showing the interface between the provisional resin disk and the resin-based coating.

The SEM images of the UV resin-based coating containing DMADDM are illustrated in Figure 4 (mean ± sd; n = 5). Each specimen is presented in two magnifications (X125 and X500) showing the interface between the provisional resin disk and the resin-based coating. The average UV+ 2.5% DMADDM coating thickness was (104.8 ± 4.1) μm, the coating thickness of the UV+ 5% DMADDM was (90.98 ± 0.36) μm, the UV+ 7.5% DMADDM had a coating thickness of (91.68 ± 0.84) μm, and the UV+ 10% DMADDM had a coating thickness of (103.58 ± 1.9) μm.

### 3.4. S. mutans Biofilm Colony-Forming Units (CFU)

**Figure 5.**
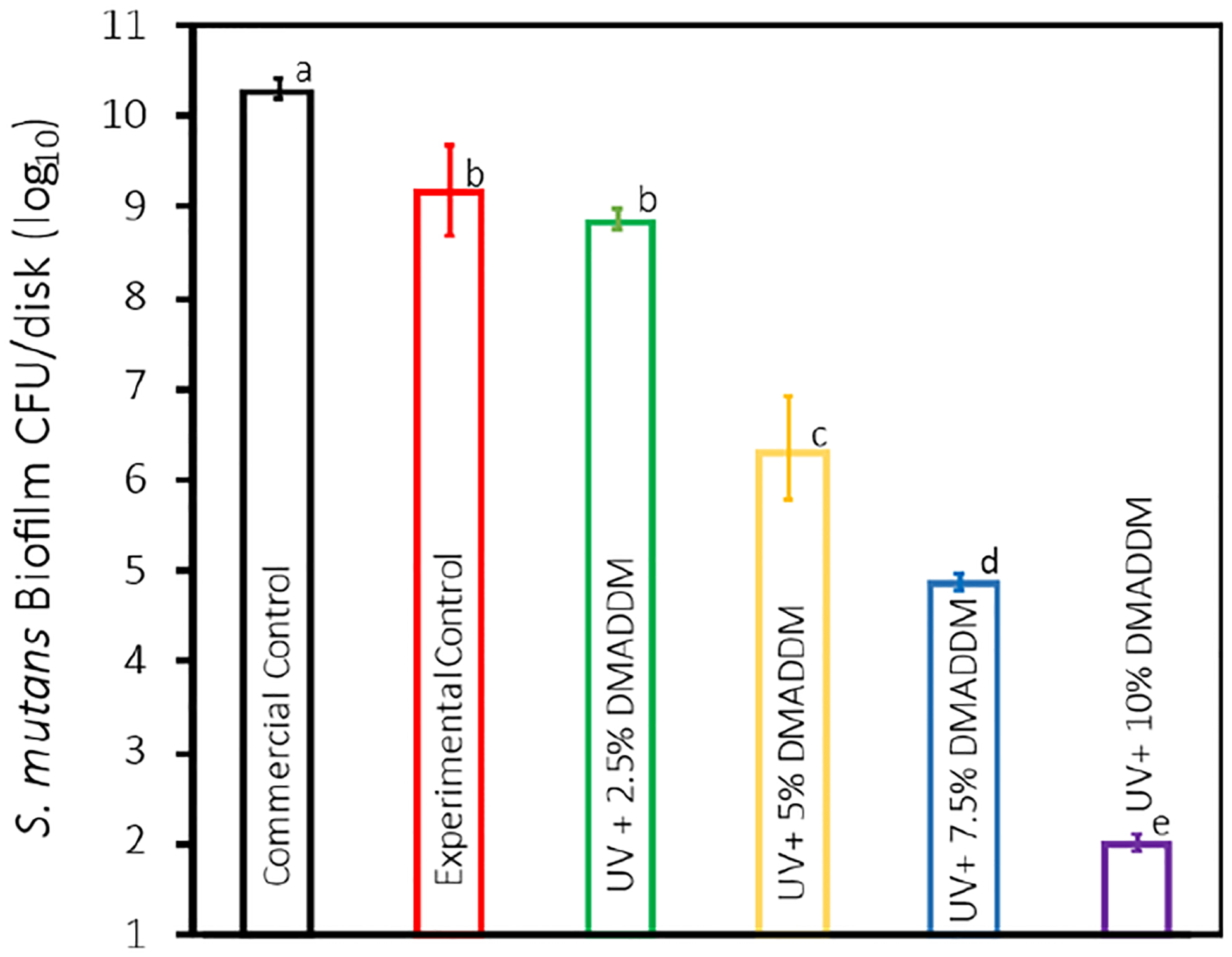
Colony-forming unit (CFU) counts of 2-day *S. mutans* biofilms adherent on the disks (mean ± sd; n = 6). Compared with the Commercial Control group, the 5% DMADDM showed a 4-log reduction (*p* < 0.01), followed by a 5-log reduction in the 7.5% DMADDM (*p* < 0.01) and an 8-log reduction in the 10% DMADDM (*p* < 0.01). Data with significant differences (*p* < 0.01) are indicated by different letters.

The results for the CFU are presented in Figure 5 (mean ± sd; n = 6). The incorporation of 5%, 7.5%, and 10% DMADDM into UV resin coating demonstrated a substantial reduction in the CFU count compared with the Commercial Control group (*p* < 0.01). The 5% DMADDM showed a 4-log reduction, followed by a 5-log reduction in the 7.5% DMADDM and an 8-log reduction in the 10% DMADDM. The difference in CFU reductions between 5%, 7.5%, and 10% DMADDM was significant (*p* < 0.01). No significant difference in CFU was noted between the Experimental Control and 2.5% DMADDM groups (*p* > 0.1).

### 3.5. Metabolic Activity of S. mutans Biofilms (MTT)

**Figure 6.**
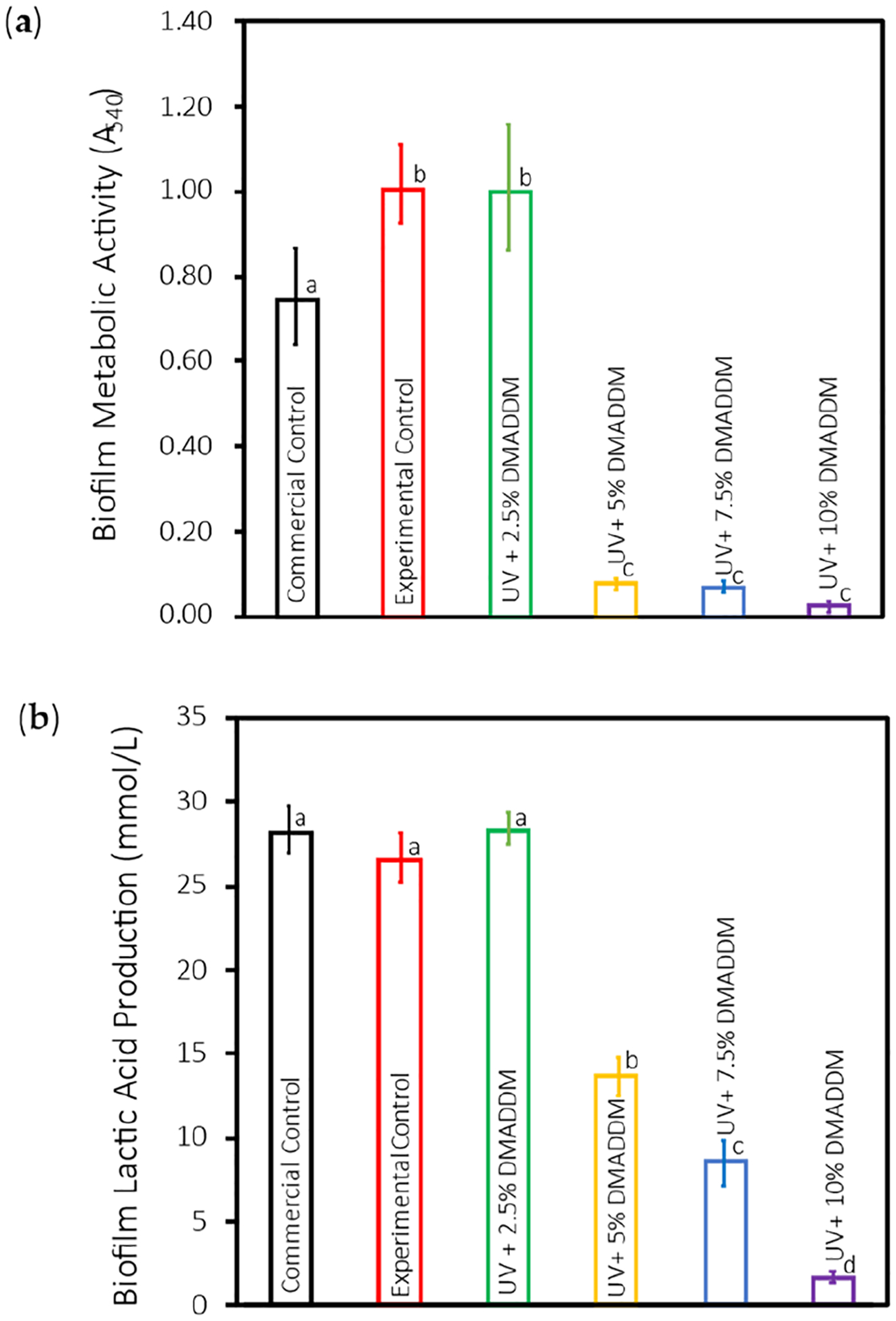
(**a**) The MTT results of *S. mutans* biofilms (mean ± sd; n = 6). The metabolic activity of the *S. mutans* biofilms significantly declined with the integration of 5%, 7.5%, and 10% DMADDM into UV resin coating compared with the Commercial Control, Experimental Control, and 2.5% DMADDM groups (*p* < 0.01). Data with significant differences (*p* < 0.01) are indicated by different letters. (**b**) The 2-day *S. mutans* biofilms’ lactic acid production (mean ± sd; n = 6). The amount of lactic acid produced was significantly higher in the Commercial Control, Experimental Control, and 2.5% DMADDM groups compared with the 5%, 7.5%, and 10% DMADDM groups (*p* < 0.01). Data with significant differences (*p* < 0.01) are indicated by different letters.

The MTT results of *S. mutans* biofilms are illustrated in Figure 6a (mean ± sd; n = 6). The metabolic activity of the *S. mutans* biofilms significantly declined with the integration of 5%, 7.5%, and 10% DMADDM into UV resin coating compared with the other groups (*p* < 0.01). No significant difference was found between the Experimental Control and 2.5% DMADDM groups in regard of MTT (*p* > 0.1).

### 3.6. Lactic Acid Production by S. mutans Biofilms

Figure 6b represents the 2-day *S. mutans* biofilms’ lactic acid production (mean ± sd; n = 6). The amount of lactic acid produced was significantly higher in the Commercial Control, Experimental Control, and 2.5% DMADDM groups compared with 5%, 7.5%, and 10% DMADDM groups (*p* < 0.01). No significant difference was found between the Commercial Control, Experimental Control, and 2.5% DMADDM groups (*p* > 0.01). Increasing the DMADDM concentration from 5% and 7.5% to 10% resulted in a significant reduction in lactic acid production, respectively (*p* < 0.01).

### 3.7. Live/Dead Staining Assay

**Figure 7.**
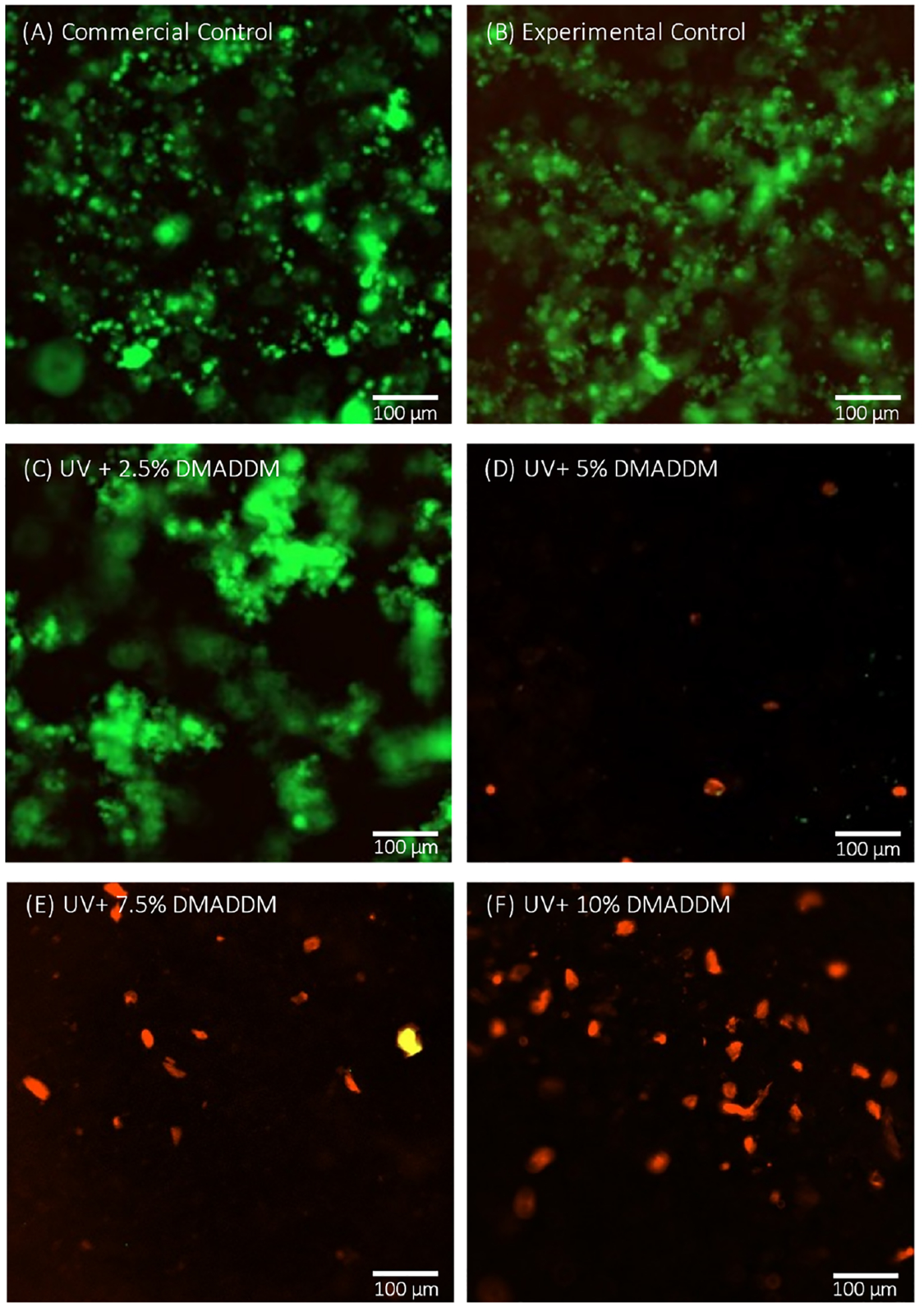
Live/dead images of 2-day *S. mutans* biofilms on the different groups. Live bacteria were stained green. Bacteria with compromised membranes were stained red. The (**A**) Commercial Control, (**B**) Experimental Control, and (**C**) UV + 2.5% DMADDM groups were primarily covered with live bacteria. The (**D**) UV+ 5% DMADDM, UV, and (**E**) 7.5% DMADDM groups were mainly covered with dead bacteria. The most potent antibacterial response was in the (**F**) UV+ 10% DMADDM group, where no sign of live bacteria (green) was visible.

Representative live/dead images of 2-day *S. mutans* biofilms on the different groups are shown in Figure 7. The Commercial Control and Experimental Control groups were primarily covered with live bacteria (green), with comparable results to the UV + 2.5% DMADDM group. In contrast, groups with 5% DMADDM and more mainly had bacteria with impaired membranes (red), with the most potent antibacterial response in the UV+ 10% DMADDM group, where no sign of live bacteria (green) was visible.

### 3.8. SEM Observation of S. mutans Biofilms

**Figure 8.**
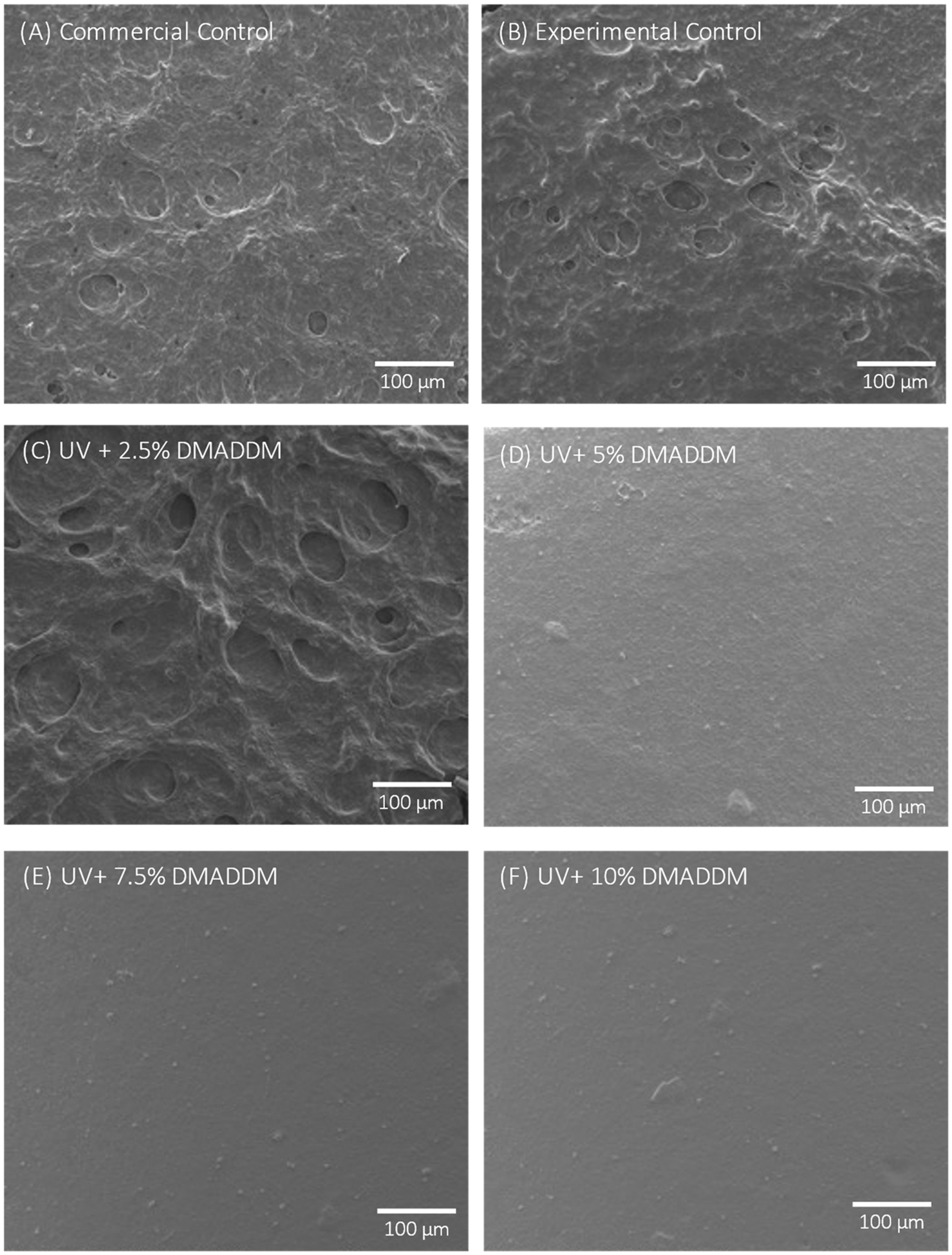
SEM images of resin specimens with a 48 h *S. mutans* biofilm. The (**A**) Commercial Control, (**B**) Experimental Control, and (**C**) UV + 2.5% DMADDM groups showed a highly complex and dense biofilm. The (**D**) UV+ 5% DMADDM, UV, (**E**) 7.5% DMADDM, and (**F**) UV+ 10% DMADDM groups effectively reduced biofilm formation, with few noticeable colonies on the surfaces.

SEM images of resin specimens with a 48 h *S. mutans* biofilm of the scanned groups are shown in Figure 8. The Commercial Control, Experimental Control, and UV + 2.5% DMADDM groups showed a highly complex and dense biofilm. Conversely, the UV+ 5% DMADDM, UV+ 7.5% DMADDM, and UV+ 10% DMADDM groups had a potent antibacterial response, yielding minimal adherence of the *S. mutans* biofilms, with few noticeable colonies on the surfaces.

### 3.9. Cytotoxicity of Human Gingival Fibroblasts

**Figure 9.**
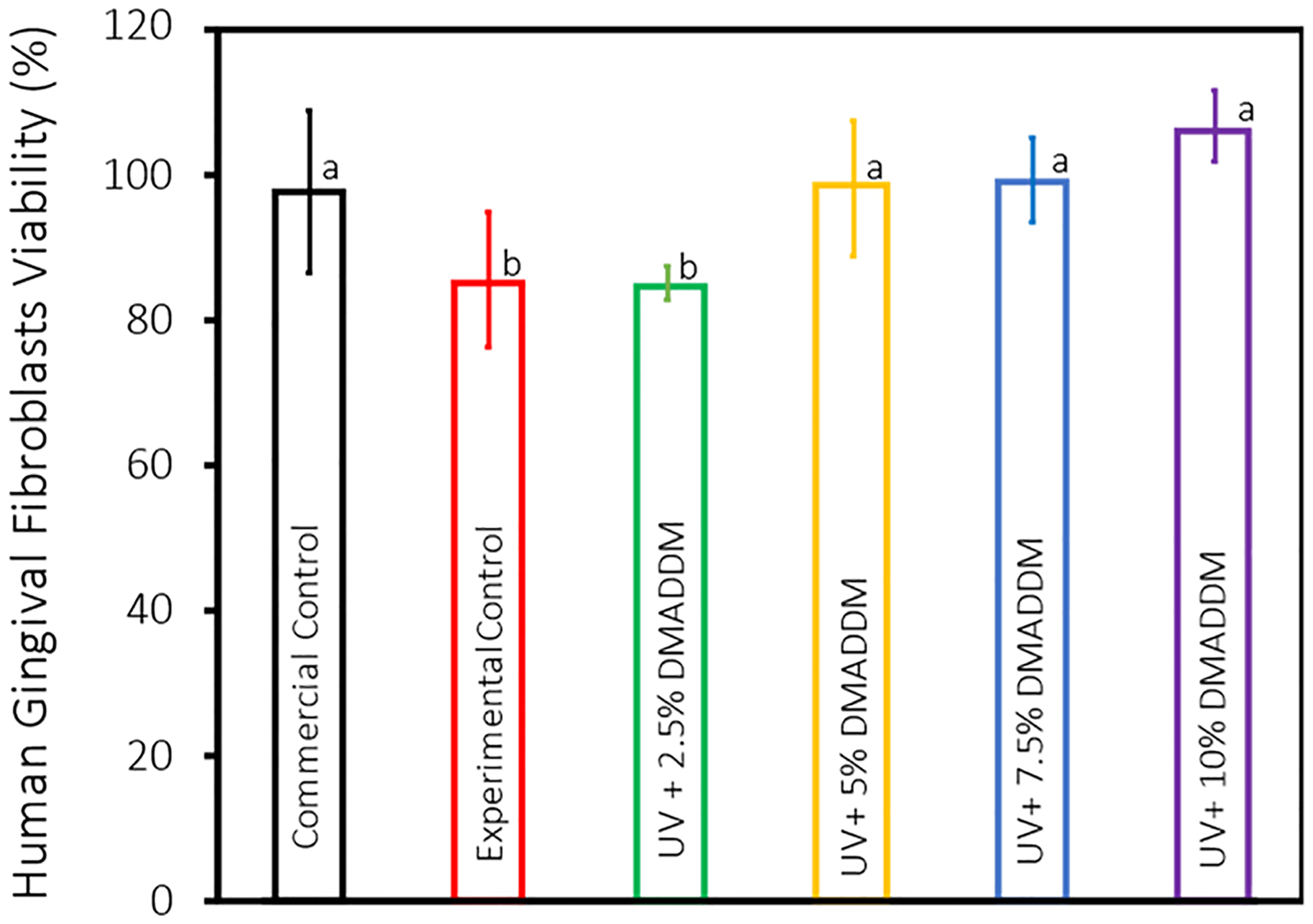
The viability of human gingival fibroblasts toward the UV resin-based coating (mean ± sd; n = 3). None of the investigated groups displayed signs of cytotoxicity affecting HGFs. Data with significant differences are indicated by different letters (*p* < 0.01).

The cytotoxicity of the UV resin-based coating was evaluated by measuring the viability of HGFs after being subjected to the resin-based coatings’ extracts, as shown in Figure 9 (mean ± sd; n = 3). All of the investigated groups had viability of >82%, indicating no cytotoxicity toward HGFs (*p* > 0.05).

## 4. Discussion

This study investigated, for the first time, a new approach of enhancing the performance of provisional crowns by developing a novel resin-based provisional crown coating material, incorporating DMADDM to inhibit secondary caries around the gingival margins of provisional crowns and exploring its antibacterial efficacy and cytocompatibility. The experimental results demonstrate the potential of DMADDM-modified coatings in reducing bacterial viability and biofilm formation without compromising the biocompatibility or surface characteristics of the material.

Secondary caries is a prevalent cause of restorative failures [47–49]. Generally, treating secondary caries might result in the additional loss of dental tissues and can potentially weaken the affected tooth [50,51]. Secondary caries can be defined as new carious lesions that develop at the margins of existing restorations [52]. The etiology of secondary caries is considered by most to be similar to the etiology of primary caries, since it is essentially a bacterial infectious disease. Nevertheless, the initial demineralization progression is merely the outcome of a complex interaction in a multi-factorial process. This interaction is undoubtedly further complicated by the presence of restorative materials in the oral environment. Primary caries is characterized by a cariogenic attack that is confined to the tooth surface, whereas in secondary caries it also affects the tooth-restoration contact. Furthermore, the restorative material has the capacity to interact with many elements that are associated with the demineralization process and can therefore be considered an extra factor [50,53].

Surface roughness and hydrophilicity are critical factors influencing bacterial adhesion and biofilm formation on dental material [19–21,54]. All groups, including the Commercial Control, Experimental Control, and DMADDM-modified coatings, exhibited similar surface roughness values (*p* > 0.05), suggesting that the incorporation of DMADDM did not negatively affect the surface texture of the material. Furthermore, all specimens evaluated in this study had an average surface roughness (Ra) of 0.19 μm or less; it was discovered that Ra value of 0.2 μm is thought to be a threshold beyond which biofilm adherence is much decreased and the surface is considered smooth [13,55]. Compared to the commercial controls, the resin-based coatings achieved low surface roughness values with fewer polishing steps, making the novel coating advantageous in clinical practices from a time-saving perspective.

Water contact angles were calculated as a qualitative measure of surface hydrophobicity, with an angle below 65° demonstrating a surface that is more hydrophilic [56]. While some studies [25,28,57,58] found that bacteria favored adherence to increased hydrophilic surfaces, other studies [59–61] did not report decreased bacterial adhesion on more hydrophobic surfaces, and still other studies did not find any significant correlation between bacterial adhesion and the hydrophobicity of a material [62,63]. Hydrophobic bacteria have a greater tendency to adhere to hydrophobic surfaces, while hydrophilic bacteria have a lower affinity for adhesion to hydrophobic surfaces [64]. *S. mutans* was observed to have a high level of hydrophobicity [64], which may account for the reduced adhesion seen in the present study to the slightly hydrophilic resin-based coatings. The contact angle results showed that while there was a significant difference in hydrophilicity between the lower and higher concentrations of DMADDM, the overall hydrophilicity did not adversely impact the coating’s performance. The hydrophilicity of dental materials can influence biofilm formation; however, the antibacterial properties of DMADDM seem to outweigh any potential increase in bacterial adhesion due to hydrophilicity. This balance between maintaining favorable surface properties and enhancing antibacterial efficacy is crucial for the success of provisional crown materials.

Regarding the contact angle of the uncured UV resin-based coatings in this study, no significant difference was seen among the experimental groups (*p* > 0.01); all the coatings showed excellent hydrophilicity. The hydrophilic coating’s increased wetting ability for provisional crowns allows for faster and easier application, which is desirable.

One of the primary cariogenic organisms in the formation of secondary caries, *S. mutans*, was chosen for this investigation to evaluate the antibacterial efficiency of the novel resin-based provisional crown coating [65]. The CFU counts confirmed the significant antibacterial activity of the DMADDM-modified coatings, showing a significant reduction in bacterial colonies with increasing DMADDM concentration. The 10% DMADDM group exhibited the highest antibacterial effect, achieving an 8-log reduction in CFU compared to the Commercial Control (*p* < 0.01) group. This significant reduction in bacterial load suggests that DMADDM can effectively inhibit the colonization and proliferation of *S. mutans*, thus potentially reducing the risk of secondary caries during the use of provisional crowns.

The metabolic activity assay (MTT) and lactic acid production measurements provided additional evidence of the antibacterial effectiveness of DMADDM. Both assays showed significant reductions in metabolic activity and lactic acid production in the 5%, 7.5%, and 10% DMADDM groups (*p* < 0.01). Lactic acid production is a critical factor in the cariogenic potential of *S. mutans*, as it contributes to the demineralization of the tooth structure. The substantial decrease in lactic acid production with higher DMADDM concentrations indicates that the novel coating can effectively disrupt the metabolic processes of *S. mutans*, thereby mitigating the risk of acid-induced caries.

The live/dead staining assay and SEM observations further supported these observations. The results showed that the coatings with 5%, 7.5%, and 10% DMADDM concentrations had potent antibacterial effects, as evidenced by the minimal presence of live bacteria and a sparse biofilm structure compared to the Commercial Control and Experimental Control groups. SEM observations corroborated these findings, showing dense biofilms on the Commercial Control, Experimental Control, and UV+2.5% DMADDM groups, while the higher DMADDM concentrations resulted in minimal biofilm formation. The observed dose-dependent antibacterial effects align with previous studies that have reported the antibacterial efficacy of DMADDM against dental biofilms [33,35,38,39].

The antibacterial efficacy of DMADDM is further emphasized by the fact that there is no significant difference in surface roughness between the experimental groups and the Commercial Control group. Bacterial adhesion and biofilm formation are known to be influenced by surface roughness, as rougher surfaces can offer a greater number of niches for bacterial colonization [54]. Nevertheless, the observed reduction in CFU, MTT, lactic acid, and biofilm formation can be confidently attributed to the intrinsic antibacterial properties of DMADDM, rather than to any variations in surface roughness, as our results demonstrate consistent surface roughness across all groups. This supports the argument that the primary reason for the effectiveness of DMADDM-incorporated resin coating is its capacity to inhibit the growth of bacteria and the development of biofilms on the material surface, rather than physical modifications to the material’s texture.

The cytotoxicity assay using human gingival fibroblasts (HGFs) demonstrated that the DMADDM-modified coatings did not negatively affect cell viability. According to ISO 10993–5, reduction of cell viability by more than 30% is considered a cytotoxic effect. All tested groups, including those with the highest DMADDM concentration, exhibited cell viabilities of >82% (*p* > 0.05) and are considered biocompatible. This result is consistent with previous research indicating that DMADDM can be incorporated into dental materials without compromising biocompatibility [66]. The biocompatibility of provisional crowns is crucial for their clinical application, as it ensures that the material will not induce adverse tissue reactions during prolonged use [67].

This study suggests that resin-based provisional crown coatings with DMADDM can reduce the risk of secondary caries around the gingival margins of provisional crowns during extended periods of use. The coatings can inhibit *S. mutans* viability and metabolic activity, maintain biocompatibility, and have favorable surface characteristics. This coating has the potential to be used in clinical scenarios where provisional crowns are required for extended periods, such as complex occlusal adjustments or multiple-stage treatments. The coating also maintains soft tissue health and minimizes gingival inflammation.

While the results of this study are promising, there are some limitations to consider. Only a single bacterial species was examined; the effect of the coating on the aesthetic characteristics of the provisional crown specimens (color, color stability over time, light reflectivity) was not assessed. Although the study findings indicate that surface roughness and hydrophilicity did not compromise the antibacterial properties of the DMADDM-based coating, these surface properties could affect other aspects critical to the clinical longevity of dental materials. Specifically, both wear resistance and long-term stability are key factors in evaluating the material’s clinical performance. It is yet to be established how easily the coating can be dislodged from the provisional crown material through ordinary toothbrushing and whether the hydrophilicity of the coating impacts hydrolytic degradation and leaching of the antimicrobial agent over time. Future research could address the limitations mentioned above and explore the incorporation of bioactive agents that can promote gingival tissue healing and investigate the effects of the coating on other oral pathogens and multi-species biofilms including Lactobacillus or Actinomyces species as well as in vivo studies to validate the findings of the current study.

## 5. Conclusions

This study introduced a novel resin-based provisional crown coating material incorporating DMADDM, designed to inhibit secondary caries around the gingival margins of provisional crowns. The findings demonstrated that DMADDM-modified coatings significantly reduced bacterial viability and biofilm formation, particularly against *S. mutans*, without compromising biocompatibility or surface characteristics. The coatings exhibited potent antibacterial effects in a dose-dependent manner, effectively reducing metabolic activity and lactic acid production. Importantly, the DMADDM coatings maintained a favorable surface roughness, which is essential for clinical application. The study suggests that these coatings could be highly beneficial in clinical scenarios requiring extended use of provisional crowns, as they offer protection against secondary caries while preserving soft tissue health.

## Data Availability

The data presented in this study are available upon request from the corresponding authors.
